# Epidemiology and cost of herpes zoster and postherpetic neuralgia among patients treated in primary care centres in the valencian community of Spain

**DOI:** 10.1186/1471-2334-11-302

**Published:** 2011-11-01

**Authors:** Ana M Cebrián-Cuenca, Javier Díez-Domingo, María San-Martín-Rodríguez, Joan Puig-Barberá, Jorge Navarro-Pérez

**Affiliations:** 1 Centro de Salud de Ayora, Avenida Argentina, Ayora, Valencia, Spain; 2 Centro Superior de Investigaciones en Salud Pública de Valencia (CSISP), Avenida Cataluña 21, 46020 Valencia, Spain; 3 Departamento Médico Sanofi Pasteur MSD, Paseo de la Castellana 141, Madrid, Spain; 4 CIBER Epidemiology and Public Health (CIBERESP), Spain, Calle Poeta Durán y Tortajada 7-55, 46022 Valencia, Spain

## Abstract

**Background:**

Data on the epidemiology and costs related to herpes zoster (HZ) and postherpetic neuralgia (PHN) in Spain are scarce; therefore, studies are needed to evaluate the epidemiological and economic impact of HZ and its most common complication, PHN. The present study aimed to estimate the clinical and economic burden of HZ and PHN in Valencia (Spain).

**Methods:**

We prospectively analyzed the burden of HZ and PHN and their attributable costs in patients from 25 general practices in the Autonomous Community of Valencia serving 36,030 persons aged > 14 years. All patients with a clinical diagnosis of HZ who attended these centers between December 1^st ^2006 and November 30^th ^2007 were asked to participate. Patients included were followed for 1 year.

**Results:**

Of the 130 cases of HZ followed up, continued pain was experienced by 47.6% (95% confidence interval (CI) = 35.6-56.7%) at 1 month after rash onset, by 14.5% (95% CI = 7.8-1.2%) at 3 months, by 9.0% (95% CI = 3.7-14.3%) at 6 months, and by 5.9% (95% CI = 1.5-10.3%) at 12 months. The percentage of patients with PHN increased with age, from 21.4% (95% CI = 8.3-40) in patients < 50 years to 59.2% (95% CI = 44.4-74) in patients ≥ 70 years. The estimated total cost for the 130 HZ cases during the follow-up period was €49,160 ($67,349). Mean cost per patient was €378 (range 53-2,830) ($517, range 73-3,877).

**Conclusions:**

This study shows that PHN is a relatively common complication of HZ and that both conditions combined give rise to a significant clinical and economic burden for patients and providers.

## Background

Herpes zoster (HZ) is caused by the reactivation of a latent infection with varicella zoster virus (VZV) after primary chickenpox. HZ is characterized by a localized eruption of vesicular lesions following the trajectory of a sensory nerve, and by the presence of pain and inflammation of the affected nerve root [1].

The most frequent and debilitating complication of HZ is postherpetic neuralgia (PHN), defined as neuropathic pain that appears in the dermatomes affected by the VZV infection. Pain associated with PHN can be very severe and disabling and has a significant impact on a patient's quality of life [2].

Studies carried out in a number of countries have shown that the incidence and severity of both HZ and PHN increase significantly with age. The annual incidence of HZ reported in population-based studies from several countries ranged from 1.2 to 4.8 cases per 1,000 inhabitants/year. The lifetime risk of HZ can be as high as 30% and increases markedly with age, affecting 50% of people living to 85 years of age [3-11]. PHN affects 10-70% of patients with HZ. This wide range is partly due to differences in the definition of PHN used in different studies and to the age range of the study populations from which these estimates were obtained. Thus, in older patients, the prevalence of PHN among patients with HZ is likely to be closer to the upper boundary of that range [12].

In a previous study we reported an annual incidence of HZ of 4.1 per 1,000 persons > 14 years of age (95% confidence interval (CI) = 3.4-4.7) and described the clinical and epidemiological characteristics of HZ in Spain [13]. Our results were consistent with previous findings by other authors in Spain [14,15], and in other European [16-25] and non-European [8,10,11,26-28] published studies.

Data on the epidemiology of HZ and PHN in Spain, and on the associated costs are scarce; therefore, studies are needed to evaluate the epidemiological and economic impact of HZ and its most common complication, PHN. The present study aimed to estimate the clinical and economic burden of HZ and PHN in the primary health care system in Spain.

## Methods

### Design and setting

During a 1-year period (from 1^st ^December 2006 to 30^th ^November 2007), a prospective study was carried out in 25 primary care general practitioner (GP) offices of the public healthcare system of the Autonomous Community of Valencia, Spain. We selected a convenience sample of 25 GP offices from rural (n = 6), urban (n = 10) and semiurban (n = 9) areas that were considered to be representative of this Autonomous Community.

### Study population

During the study period, all patients > 14 years of age who attended the investigators' offices and were clinically diagnosed with HZ were considered to be eligible for inclusion. All patients who agreed to participate in the study signed an informed consent form prior to their participation.

For each patient, information was collected on demographic attributes, personal clinical history, clinical characteristics of the HZ episode and associated complications, medical visits and medication used to treat the episode or its complications, and work absenteeism of the patients or their carers. The information was obtained by interview with the patient and by review of their medical records. No further medical visits were planned for study purposes. Patients were telephoned the day after the diagnosis, and four times during follow-up (at 1, 3, 6 and 12 months after diagnosis). If the patients reported pain, they were contacted monthly until disappearance of the pain. In those cases where patients could not be accessed by phone, they were visited at home by the study investigators in order to establish their clinical status.

One hundred and forty six patients were diagnosed and invited to participate. Those who declined to participate (n = 16; 10.9%) were anonymously counted as HZ cases to allow a real estimate of the incidence of the disease. Of the 130 participants, 1-year complete data was obtained in 118 patients (90.8%). Thus, we had incomplete follow-up data for 12 patients (9.2%): six died because of factors unrelated to HZ, two withdrew voluntarily from the study and four patients were lost to follow-up.

### Study measures and definitions

For the purpose of the present study we considered a case of HZ as a localized eruption of vesicular lesions following the trajectory of a sensory nerve as well as pain and inflammation of the affected nerve root. This definition was purely clinical since no other confirmatory test was necessary. A diagnostic test was performed only if the GP determined that it was necessary.

We defined PHN as the presence of any pain, without specifying a numerical threshold of intensity. We believe that this definition is in accordance with most publications in this setting [27,29-32]. PHN is defined as pain that persists beyond the acute phase of an HZ episode. For this study, we considered PHN1, PHN3, PHN6 and PHN12 as pain persisting 1, 3, 6 or 12 month(s), respectively, after the onset of the rash. We defined "recurrence" as the development of cutaneous lesions on the site of a previous eruption or at a different site, which occurred beyond 3 months after the index HZ episode was diagnosed. Recurrent HZ was not counted as a new case.

Direct costs were evaluated as those derived from medical care and medications, and included: 1) the number of medical visits: primary care visits, primary care emergency consultations, specialist visits, other professional visits, hospitalizations, hospital emergencies; 2) medications prescribed and/or consumed; and 3) diagnostic tests. We included the costs arising from symptoms occurring in the time period preceding the vesicular rash, and all patients were asked, as per protocol, about these outpatient or hospital visits during the initial interview.

The unit costs for the medical care variables were obtained from the Valencian Community Official Registries (Presupuestos del Ejercicio 2008 de la Generalitat Valenciana) [33] and the cost of medications was obtained from the Vademecum^® ^2007 Edition as a reference for each medication price [34]. Indirect costs were defined as the productivity losses of patients and/or their carers, which were calculated from the number of hours of work missed related to HZ. The cost for an effective working hour in 2007 was obtained from the Spanish National Institute of Statistics [35]. For the present analysis we also took into consideration whether patients were active workers (for which 60% of the cost of medications is reimbursed by Spanish Social Security) or pensioners (for which 100% is reimbursed) [36]. All costs are presented in 2007 Euros (€).

The study was approved by the Clinical Research Ethics Committee of the Dirección General de Salud Pública/Centro Superior de Investigación en Salud Pública (CSISP) de la Comunidad Valenciana.

### Sample size calculation: Statistical analysis

Based on an expected incidence of HZ in the adult population of 0.4% and a precision of 0.08%, we calculated that a minimum population of 23,600 people > 14 years of age should be monitored [6]. The study population assigned to the 25 participating investigators represented a total of 36,030 individuals. This population comprised 21,500 patients aged 15-49 years, 4,893 patients aged 50-59 years, 4,057 patients aged 60-69 years, and 5,580 patients aged ≥ 70 years. The age and gender distribution of patients recruited in our study was similar to that observed in the general population (based on data from national Spanish registries [37]).

The percentage of patients with persisting pain during follow-up was analyzed using the Kaplan-Meier method. The incidence was calculated globally by gender and by predefined age groups (< 50 years, 50-59 years, 60-69 years, and ≥ 70 years of age). Comparisons between groups were performed using Student's t-test for continuous variables with Gaussian distribution.

Logistic regression was used to identify predictors of the occurrence of PHN at 1 and 3 months (dependent variable). We considered as covariates those variables that showed a P-value of ≤ 0.25 in the univariable analysis (presence of PHN *versus *no PHN) and those clinically relevant in previous studies (age, gender, prodromal pain, extremities localization, sacrum localization, time from symptoms onset until diagnosis, and time from rash onset until diagnosis). We used an "enter" variable selection method. We obtained the odds ratio (OR) and 95% CI. Model calibration was tested with the Hosmer-Lemeshow test. Model discrimination was evaluated with the analysis of the area under the ROC (Receiver Operating Characteristic) curve. All P-values < 0.05 were considered to be statistically significant. All statistical analyses were performed using the SPSS software package, version 17.0 (SPSS Inc., Chicago, IL, USA).

## Results

### Epidemiology of PHN

Baseline characteristics of study patients are presented in Table 1. A total of 146 HZ patients were diagnosed within the 1-year period, an annual incidence of 4.1 cases per 1,000 persons > 14 years (95 CI % = 3.4-4.7). Median age was 63.5 years (range 19-94; interquartile range 51-75 years) and the highest incidence was observed in older patients (≥ 70 years of age), namely 11.1/1,000 population.

**Table 1:** Baseline characteristics of study patients

Variable	n = 130
Age, years, median (range)	63.5 (19-94)
Female gender, n (%)	83 (63.8)
**Area of residence**	
Urban, n (%)	41 (31.5)
Semiurban, n (%)	39 (30)
Rural, n (%)	50 (38.5)
**Predisposing clinical conditions†**	60 (46.2)
Immunosuppressant* use, n (%)	3 (2.3)
Malignancy, n (%)	12 (9.2)
HIV, n (%)	0
Transplant, n (%)	1 (0.8)
Chronic disease*, n (%)	39 (30)
Trauma/burns/radiotherapy, n (%)	3 (2.3)
Other**	6 (4.6)
Not reflected in clinical history, n (%)	2 (1.5)
**Complications†, n (%)**	31 (23.8)
Ocular, n (%)	11 (8.5)
Bacterial superinfection, n (%)	7 (5.4)
Dysgeusia, n (%)	4 (3.1)
Hypoacusia, vertigo, tinnitus, n (%)	2 (1.5)
Dissemination, n (%)	2 (1.5)
Involvement of other organs, n (%)	1 (0.8)
Other‡, n (%)	12 (9.2)
**Use of antiviral agents, n (%)**	119 (91.5)
**Time elapsed from symptom onset to diagnosis, days (SD)**	6.3 (5.8)

SD: Standard deviation.

HIV: Human immunodeficiency virus.

* Administration of oral or systemic corticosteroids or chemotherapy treatment.

† Each patient may have more than one baseline clinical condition/complication.

* Chronic disease was defined as diabetes mellitus, chronic obstructive pulmonary disease, moderate or severe asthma, rheumatoid arthritis, chronic liver disease, chronic kidney disease, congenital heart disease, and systemic lupus erythematosus.

** Other risk factors refer to multisystem atrophy, major depression, postsurgical stress, influenza, and polysubstance abuse.

‡ Other complications refer to lymphadenopathy, activity limitation, fungal infection in the herpes zoster area, allergic reactions to treatment, weight loss, anorexia, vomiting, and diarrhea.

**Figure 1:**
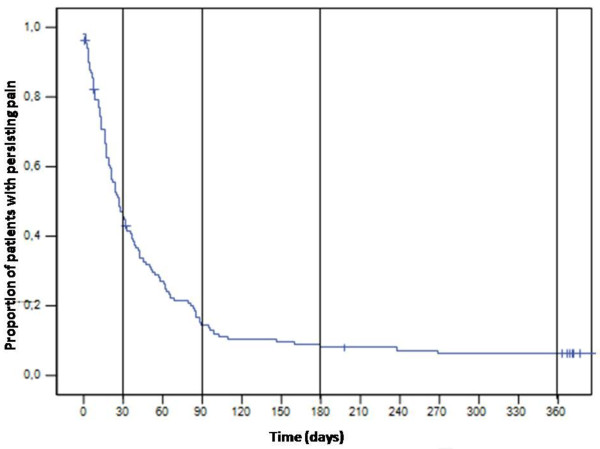
**Kaplan-Meier method showing the percentage of patients with persisting pain during the follow-up**.

**Figure 2:**
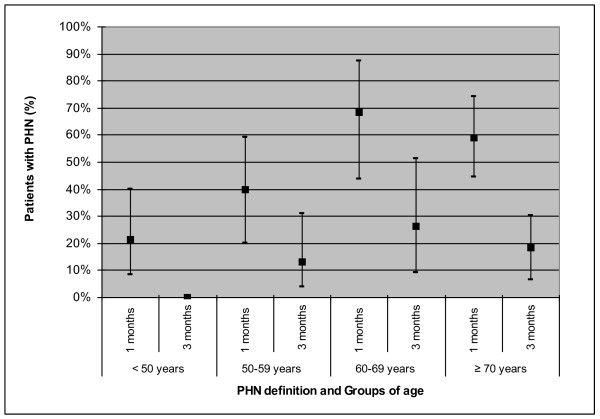
**Percentage of patients developing postherpetic neuralgia (PHN) at 1 and 3 months per age groups (95% confidence intervals are presented)**.

In the present study, only one case (1/130) of recurrence at the same site was reported at 7 months. No recurrences (0/130) at different sites were observed. Pain continued to be experienced by 47.6% of patients at 1 month after rash onset (95% CI = 35.6-56.7%), by 14.5% (95% CI = 7.8-21.2%), at 3 months, by 9.0% (95% CI = 3.7-14.3%) at 6 months, and by 5.9% (95% CI = 1.5-10.3%) at 12 months (Figure 1). The median duration of pain was 26.5 days (95% CI = 20-34). Figure 2 shows the percentage of patients with PHN at 1 month and 3 months in each age group. The mean age of patients who fulfilled the PHN definition at 1 month was 66.7 years, and 61.7% of the patients were female. In the case of PHN at 3 months, the mean age was 70.1 years, and 50% of the patients with PHN were female. The percentage of patients developing PHN (at 1 month) increased with age from 21.4% (95% CI = 8.3-40) in patients < 50 years to 59.2% (95% CI = 44.4-74) in patients ≥ 70 years (Figure 2).

In the multivariate model, age (years) (OR = 1.04, P < 0.01) and time interval (days) between symptom onset and clinical diagnosis (OR = 1.11, P < 0.01) were both independently associated with PHN at 1 month. At 3 months, only age (OR = 1.04, P = 0.03) was found to be associated with PHN. However, we did not observe a significant association of gender or antiviral drug use (both P > 0.05) with the occurrence of PHN.

### Health care and medication costs of HZ disease

During the follow-up period, 130 cases of HZ generated a total of 308 primary care visits (mean per patient = 2.37; standard deviation (SD) = 1.99), 86 visits to the Emergency Room in Primary Care Centers (mean per patient = 0.66; SD = 1.04), 38 Specialist visits (mean per patient = 0.29; SD = 1.08), 40 other professional (nurse, physiotherapist and alternative medical practitioner) visits (mean per patient = 0.31; SD = 2.82), 23 hospital Emergency Room visits (for first medical contact for the diagnosis of HZ before being attending the GP office, and for episodes of severe pain) (mean per patient = 0.18; SD = 0.52), and 29 diagnostic tests (including X-ray studies (thorax and abdomen), blood and urine tests, electrocardiograms and ultrasound scans in patients with pain but no cutaneous manifestations, before the HZ rash appeared) (mean per patient = 0.22; SD = 0.64). No confirmation molecular tests were performed. No hospitalizations for HZ disease were observed during the study follow-up.

The total cost for the management of the 130 patients included in the analysis during the 1-year follow-up period was €40,234 (US$55,120) for a Third-Party Payer (TPP) perspective (Spanish Social Security), and €49,160 ($67,349) from a societal perspective, yielding an average cost per patient of €309, SD = 308 ($423, SD = 422) and €378, SD = 431 ($518, SD = 590), respectively. The most important factor that contributed to the economic burden of disease (Table 2) was medication (33% and 37% of the cost from the TPP perspective and the societal perspective, respectively) followed by GP visits (29% and 23% of the cost from the TPP perspective and the societal perspective, respectively), and Emergency in Primary Care visits (18% and 15% of the cost from the National Payer perspective and the societal perspective, respectively) (Table 2).

**Table 2:** Total cost* for patients included in the study with diagnosis of herpes zoster

	TPP perspective**				Societal perspective‡			
**Direct costsϕ**	**Total**	**Mean**	**SD**	**%**	**Total**	**Mean**	**SD**	**%**

Primary care visits	11,521.24	88.62	56.14	28.64%	11,521.24	88.62	56.14	23.44%

Primary care emergencies	7,178.42	55.22	86.68	17.84%	7,178.42	55.22	86.68	14.60%

Specialist visits	1,726.48	13.28	41.76	4.29%	1,726.48	13.28	41.76	3.51%

Other professional visits ^□^	662.80	5.10	46.72	1.65%	662.80	5.10	46.72	1.35%

Hospitalizations	0	0	0	0	0	0	0	0

Hospital emergencies	2,369.92	18.23	53.62	5.89%	2,369.92	18.23	53.62	4.82%

Diagnostic tests	625.25	4.81	13.92	1.55%	625.25	4.81	13.92	1.27%

Medication	13,443.62	103.41	132.38	33.41%	18,115.26	139.35	138.26	36.85%

**Indirect costs**#								

Absenteeism from work	2,706.18	20.82	137.74	6.73%	6,887.35	52.98	316.86	14.01%

Carers †	0	0	0	0	73.14	0.56	6.41	0.15%

* In 2007 euros.

^□ ^Other professionals: physiotherapist, nurses and alternative medical practitioners.

† Carers: those persons or relatives who took care of herpes zoster patients.

** TPP Perspective (Third-Party Payer = Spanish Social Security): public medical visits + diagnostic test + percentage that Spanish National Health System pays for prescriptions.

‡ Societal perspective: TPP perspective items + percentage that patient pay for prescription + private medical visits + medication not included in the Spanish National System + cost for lost hours of work.

ϕ Direct costs: medical care and medications, and included: 1) the number of medical visits: primary care visits, primary care emergency consultations, specialist visits, other professional visits, hospitalizations, hospital emergencies; 2) medications prescribed and/or consumed and 3) diagnostic tests.

# Indirect costs: productivity losses of patients and/or their carers, calculated from the number of hours of work missed due to herpes zoster.

Ninety-one percent of patients were prescribed antivirals, representing 77% of the total cost of medication. Other medications used were anticonvulsants (12.2%), analgesics (4.7%), antidepressants (0.6%), opioids (0.5%) and corticoids (0.4%), accounting all together for 18.4% of the total costs of medications. Total and mean costs per age groups are summarized in Table 3.

**Table 3:** Total cost* for patients included in the study with diagnosis of herpes zoster by age subgroups

	TPP perspective**			**Societal perspective**‡		
Age	**Total**	**Mean**	**SD**	**Total**	**Mean**	**SD**

< 50 years	6,131.76	211.44	166.60	9,059.86	312.41	360.52

50-59 years	7,170.86	239.03	231.48	11,235.57	374.52	485.30

60-69 years	8,652.79	432.64	434.52	10,426.75	521.34	626.65

≥ 70 years	18,278.50	358.40	329.37	18,437.69	361.52	330.04

Total	40,233.91	309.49	307.47	49,159.86	378.15	430.67

	P = 0.027			P = 0.401		

* In 2007 Euros.

** TPP Perspective (Third-Party Payer = Spanish Social Security): public medical visits + diagnostic test + percentage that Spanish National Health System pays for prescriptions.

‡ Societal perspective: TPP perspective items + percentage that patient pay for prescription + private medical visits + medication not included in the Spanish National System + cost for lost hours of work.

Our study patients lost a total of 565 hours of work (mean per patient = 4.35; SD = 25.99) and their carers lost 6 hours of work (mean per patient = 0.05; SD = 0.53).

### Cost of PHN1

Total cost of the management of patients with PHN1 was significantly (P < 0.001) higher (total cost, €32,974 ($45,110); mean per patient, €549 ($752), SD = €580 ($795)) compared with the cost for patients without PHN (total costs, €16,186 ($22,175)); mean per patient, €231 ($316); SD = €107 ($147)). The most important factor that contributed to the cost was medication (€10,923 ($14,964); mean per patient, €182 ($249), SD = €182 ($249) followed by GP visits (€6,686 ($9,160); mean per patient, €111 ($152), SD = €69 ($95)) and Emergency in Primary Care visits (€4,173 ($5,717); mean per patient, €70 ($96), SD = €111 ($152)) (Table 4).

**Table 4:** Cost* of herpes zoster and postherpetic neuralgia at 1 month

		TPP perspective**			Societal perspective‡		
**Direct costsϕ**		**Total**	**Mean**	**SD**	**Total**	**Mean**	**SD**

Primary care visits	With PHN	6,686.34	111.44	69.02	6,686.34	111.44	69.02
	
	Without PHN	4,834.90	69.07	31.29	4,834.90	69.07	31.29

Primary care emergencies	With PHN	4,173.50	69.56	111.00	4,173.50	69.56	111.00
	
	Without PHN	3,004.92	42.93	56.38	3,004.92	42.93	56.38

Specialist visits	With PHN	1,301.86	21.70	57.33	1,301.86	21.70	57.33

	Without PHN	424.62	6.07	18.33	424.62	6.07	18.33
	
Other professional visits ^□^	With PHN	629.66	10.49	68.62	629.66	10.49	68.62

	Without PHN	33.14	0.47	2.78	33.14	0.47	2.78
	
Hospitalizations	With PHN	0	0	--	0	0	--

	Without PHN	0	0	--	0	0	--
	
Hospital emergencies	With PHN	2,060.80	34.35	72.65	2,060.80	34.35	72.65

	Without PHN	309.12	4.42	21.02	309.12	4.42	21.02
	
Diagnostic tests	With PHN	438.75	7.31	17.35	438.75	7.31	17.35

	Without PHN	186.50	2.66	9.73	186.50	2.66	9.73
	
Medication	With PHN	8,626.16	143.77	177.84	10,923.44	182.06	182.45

	Without PHN	4,817.45	68.82	55.59	7,191.82	102.74	65.79
	
**Indirect cost**#							

Absenteeism from work	With PHN	2,706.18	45.10	200.92	6,686.22	111.44	461.46
	
	Without PHN	0	0	0	201.14	2.87	9.94

Carers †	With PHN	0	0	0	73.14	1.22	9.44
	
	Without PHN	0	0	0	0	0	0

* In 2007 euros.

^□ ^Other professionals: physiotherapist, nurses and alternative medical practitioners.

† Carers: those persons or relatives who took care of herpes zoster patients.

** TPP Perspective (Third-Party Payer = Spanish Social Security): public medical visits + diagnostic test + percentage that Spanish National Health System pays for prescriptions.

‡ Societal perspective: TPP perspective items + percentage that patient pay for prescription + private medical visits + medication not included in the Spanish National System + cost for lost hours of work.

### Cost of PHN3

Total cost of management of patients with PHN3 was significantly higher (P < 0.001) (total costs, €14,786 ($20,257); mean per patient, €821 ($1,125), SD = €681 ($941)) compared with the cost for patients without PHN (total costs, €34,373 ($47,091); mean per patient, €307 ($421), SD = €328 ($449)). The most important factor that contributed to the cost was medication (€5,730 ($7,850); mean per patient, €318 ($436), SD = €280 ($384)) followed by Emergency in Primary Care visits (€2,421 ($3,317); mean per patient, €134 ($184), SD = €165 ($226)) and GP visits (€2,393 ($3,278); mean per patient, €133 ($182), SD = €91 ($125)) (Table 5). None of the study patients was treated by pain clinics during the one year follow-up. Table 6 presents detailed information regarding the consumption of resources by patients with PHN1 and PHN3.

**Table 5:** Cost* of herpes zoster and postherpetic neuralgia at 3 months

		TPP perspective			Societal perspective		
**Direct costs**							

		**Total**	**Mean**	**SD**	**Total**	**Mean**	**SD**

**Primary care visits**	With PHN	2,393.54	132.97	90.73	2,393.54	132.97	90.73
	
	Without PHN	9,127.70	81.50	45.08	9,127.70	81.50	45.08

**Primary care emergencies**	With PHN	2,420.63	134.48	164.81	2,420.63	134.48	164.81
	
	Without PHN	4,757.79	42.48	58.21	4,757.79	42.48	58.21

**Specialist visits**	With PHN	525.41	29.19	62.90	525.41	29.19	62.90
	
	Without PHN	1,201.07	10.72	37.06	1,201.07	10.72	37.06

**Other professional visits**	With PHN	33.14	1.84	5.36	33.14	1.84	5.36
	
	Without PHN	629.66	5.62	50.31	629.66	5.62	50.31

**Hospitalizations**	With PHN	0	0	--	0	0	--
	
	Without PHN	0	0	--	0	0	--

**Hospital emergencies**	With PHN	1,133.44	62.97	100.83	1,133.44	62.97	100.83
	
	Without PHN	1,236.48	11.04	37.52	1,236.48	11.04	37.52

**Diagnostic tests**	With PHN	209.85	11.66	17.72	209.85	11.66	17.72
	
	Without PHN	415.40	3.71	12.97	415.40	3.71	12.97

**Medication**	With PHN	4,718.82	262.16	282.99	5,730.03	318.34	279.66
	
	Without PHN	8,724.80	77.90	57.95	12,385.23	110.58	64.87

**Indirect costs**							

**Absenteeism from work**	With PHN	965.45	53.64	227.56	2,340.48	130.03	515.71
	
	Without PHN	1,740.73	15.54	117.96	4,546.87	40.60	273.55

**Carers**	With PHN	0	0	0	0	0	0
	
	Without PHN	0	0	0	73.14	0.65	6.91

* In 2007 Euros.

**Table 6:** Resource consumption PHN* at 1 and 3 months

		*PHN AT 1 MONTH*			*PHN AT 3 MONTHS*		
		**Total**	**Mean per patient**	**SD**	**Total**	**Mean per patient**	**SD**

**Primary care visits**	With PHN	192	3.20	2.48	72	4	3.27
	
	Without PHN	116	1.66	1.02	236	2.11	1.57

**Primary care emergencies**	With PHN	50	0.83	1.33	29	1.61	1.97
	
	Without PHN	36	0.51	0.68	57	0.51	0.7

**Specialist visits**	With PHN	31	0.52	1.53	12	0.67	1.68
	
	Without PHN	7	0.10	0.30	26	0.23	0.95

**Other professional visits**^□^	With PHN	38	0.63	4.14	2	0.11	0.32
	
	Without PHN	2	0.03	0.17	38	0.34	3.04

**Hospitalizations**	With PHN	0	0	--	0	0	-
	
	Without PHN	0	0	--	0	0	-

**Hospital emergencies**	With PHN	20	0.33	0.71	11	0.61	0.98
	
	Without PHN	3	0.04	0.20	12	0.11	0.36

**Diagnostic tests**	With PHN	21	0.35	0.80	10	0.56	0.78
	
	Without PHN	8	0.11	0.44	19	0.17	0.6

* PHN: Postherpetic neuralgia.

^□ ^Other professionals: physiotherapist, nurses and alternative medical practitioners.

## Discussion

No previous study has provided current HZ epidemiological data and a detailed estimate of the associated costs in Spain. It is important to highlight that our study was prospectively designed and patients' symptoms were monitored periodically by phone until PHN resolution. As mentioned above, no medical visits were planned for study purposes in order to allow for a more accurate estimate of healthcare resource consumption associated with HZ management. Moreover, this is the first study in Spain that provides information about the cost of HZ and PHN on an outpatient basis. Others have reported the cost of HZ in patients admitted to hospital [38].

In our study the 1-year incidence of HZ was 4.1 per 1000 persons (95% CI = 3.4-4.7) in those aged > 14 years. The incidence of PHN was 47.6% at 1 month. This percentage was higher than the figures reported in retrospective studies in Europe (14.3% [all ages of subjects] [39] to 19.5% [≥ 50 years of age] [24]) but similar to that in a prospective study (51.2%) recently published in a Mediterranean country [40]. In the USA, a groundbreaking study by Yawn [27] reported that the incidence of HZ in subjects > 22 years in the US was 3.6 per 1000 person-years (95% CI = 3.4-3.7). The incidence of PHN in the same population was 82% when defined as pain of at least 1 month duration. In the case of 3-month PHN (PHN3), our results (14.5%) were similar to those reported in other observational studies in Europe [23,24,40], and also to those reported in the USA [4,27].

Although differences were observed with regard to PHN1 between our study and that of Yawn [27], the incidence of HZ and PHN at 3 months was similar though determined in slightly different populations (> 22 vs. > 14 years). We do not have a reason for the 1-month difference as both study populations showed a similar distribution of age and gender, both of which have an effect on the incidence of PHN. Nevertheless, the different study designs might have an impact in this regard. The study by Yawn [27] was retrospective and the present investigation was prospective to better estimate the occurrence of PHN.

The main risk factor for developing PHN among immunocompetent subjects in our study was age. An increase in 1 year of age yielded a 4.2% increase in the risk of PHN at 1 and 3 months. This finding has been previously observed by other authors [23,24].

There is controversy as to whether gender can be considered an actual risk factor for PHN as some studies found an association [24,39] while others did not [23]. Differences between genders, regarding perception and response to pain, may explain the disparities [41], or a higher GP attendance by women may play a part.

In our study the overall percentage of patients treated with antivirals was high, namely 91%, when compared with the proportion of patients (58.8%) in whom treatment was initiated within 72 hours of the onset of rash (following the current treatment recommendations). However, this proportion was not substantially different to that observed in other previously published studies, such as the Oxman study [4]. In that study, the percentage of patients treated with antiviral drugs ranged from 85.7% to 87.3%. Differences in study design might have influenced the small differences between the studies.

In our study the prescription of antiviral therapy was not associated with the occurrence of PHN, although it should be stressed that a high percentage of patients (41.2%) received antivirals beyond 72 hours after rash onset. After adjusting for other co-variates, such as age and presence of chronic illness, it was not found that antivirals would act as a preventive measure for the development of PHN.

This study also provides a recent estimate of the economic burden of HZ and PHN in a developed country. Given that the size of Spanish population age > 14 years in 2007 was 38,443,352 inhabitants, and that the prevalence of HZ was 4.1 cases per 1,000 persons > 14 years (95% CI = 3.4-4.7), we estimate a national cost of €59.6 million per year (95% CI = 49.4-68.3), which represents 0.06‰ of the 2007 gross national product (GNP).

In comparing with other European studies, our investigation found the cost of HZ to be €378 per patient, roughly double the cost reported by Gautier [24] (103 GB Pounds [£] equivalent to €151), but was lower than those reported by Davies [42] (£770, equivalent to €1132), Edmunds [43] (£306 equivalent to €450) and Scott [23] (£524, equivalent to €770). The high cost reported by Davies [42] could be due to the fact that the patients attended a tertiary referral center with a specialist pain clinic. We report a cost per PHN episode of 549 € using the 1-month definition, similar to that reported by Gautier [24] (£341, equivalent to €501). Applying the 3-month PHN definition, we report a total cost equal to €821 per patient while it was of £397 (€584) in Gautier's study [24].

In the USA, the comparable study by Yawn [44] found that the mean HZ-attributable cost was $782 (€531) for those patients in an outpatient setting. In our study (no hospitalizations were recorded) the mean cost was €378, significantly lower than that reported in the Yawn study [44]. For those patients who developed PHN, a mean cost of $4,388 per patient (€3203) was obtained in the Yawn study [44]. Again, this figure was substantially higher than our estimate (€821). Noteworthy in the Yawn's study there were 66 HZ-related hospitalizations, (with a mean length of stay of 5.1 days) compared to none hospitalizations in our study. This could explain, at least partially, the observed differences.

Comparisons between different studies from different countries are controversial due to substantial disparities in economic, demographic, cultural and institutional (health sector) structures [45]. Moreover, the different rate of antiviral prescribing (50% in the Gautier [24] study *vs*. 91% in the present study) might have accounted for some of the differences in the total cost between the studies. This issue is in contrast with the current recommendations of clinical consensus reports and needs to be studied in detail separately [46].

In line with Gautier's study in Europe [24] and Yawn's study in the USA [44], the most important factor that contributed to the economic burden was medication, followed by general practitioner visits.

HZ and PHN have an important impact for the Spanish Health Service, society and the individual. Current therapies and options do not completely alleviate the acute pain, and fail to prevent PHN, and thus provides preventive measures such as vaccination may be warranted. Zoster vaccine has been shown to reduce the burden of HZ by decreasing the incidence and symptom severity, and it has also been shown to reduce the incidence of PHN compared with placebo [4].

Our study has some limitations. First, those patients with HZ who may have gone to a private practitioner might not be detected in our study, leading to an underestimate of the disease; however, it can be presumed that this proportion of patients would be small given the free health care provision in Spain and the reimbursement of drugs prescribed in public GP offices. In Spain many patients directly attend Accident & Emergency (A&E) departments when they perceive a disease as "serious". This might have underestimated our incidence result. Any way, we consider that this underestimation should not be of considerable importance as in the Valencian Community, usually patients diagnosed by the A&E departments physicians or private specialists, in addition, attend to their primary care GP for prescription and reimbursement of medication, thus being susceptible to be recruited in the study.

Second, our study is not a population-based study but is based on a convenience sample of primary healthcare GP offices, so extrapolation of our results to the general adult population should be made with caution. Finally, in our study the costs related to working time lost showed a wide range of variation as indicated by a high standard deviation, thus making interpretation difficult.

## Conclusions

HZ and PHN are prevalent diseases in Spain whose incidence increases with age. HZ and PHN have a significant clinical and economic burden. Randomized controlled trials would be appropriate and necessary to investigate the role of antiviral therapy in preventing PHN when prescribed more than 72 hours after rash onset.

## Abbreviations

HZ: herpes zoster; VZV: varicella zoster virus; PHN: postherpetic neuralgia; CI: confidence interval; GP: general practitioner PHN1: PHN at 1 month; PHN3: PHN at 3 months; PHN6: PHN at 6 months; PHN12: PHN at 12 months; €: euros; $: US dollars; OR: odds ratio; SD: standard deviation; TPP: Third-Party Payer; £: GB pounds; GPN: gross national product; A&E: Accident and emergency departments.

## Competing interests

Unrestricted grants from Conselleria de Sanitat of the Generalitat Valenciana reference number: A.P. 021/06 (Diari official de la Generalitat Valenciana (DOGV) number 5.373 from 24/October/2006) and Sanofi Pasteur MSD were used to support this research, but this financial support did not influence the analysis or interpretation of the data. JDD has received research funding from Sanofi Pasteur MSD and GlaxoSmithKline and also payments for attending advisory board meetings. JPB is principal investigator in clinical trials with GlaxoSmithKline vaccines. MSR is a staff member of Sanofi Pasteur MSD. The following authors declare that they have no competing interests: ACC and JNP.

## Authors' contributions

ACC and JDD contributed to protocol preparation, patient recruitment, data collection, data analysis, results discussion and manuscript preparation. JPB and JNP contributed to results discussion and the critical revision of the manuscript. MSR contributed to data analysis and manuscript preparation. All authors read and approved the final manuscript.

## Pre-publication history

The pre-publication history for this paper can be accessed here:

http://www.biomedcentral.com/1471-2334/11/302/prepub
